# Resistance Patterns of *Neisseria gonorrhoeae* in PLHIV: A Cross-Sectional Study from the Republic of Cyprus, 2015–2023

**DOI:** 10.3390/antibiotics14060589

**Published:** 2025-06-07

**Authors:** Michaela Takos, George Siakallis, Annalisa Quattrocchi, Maria Alexandrou, Panagiota Papadamou, Loukia Panagiotou, Danny Alon-Ellenbogen

**Affiliations:** 1 Department of Basic and Clinical Sciences, University of Nicosia Medical School, Nicosia 2417, Cyprus; siakallis.g@med.unic.ac.cy (G.S.); alon-ellenbogen.d@unic.ac.cy (D.A.-E.); 2 School of Health and Medical Sciences, St George’s University of London, London SW17 0RE, UK; 3 Grigorios HIV Reference Clinic, Larnaca General Hospital, Larnaca 6043, Cyprus; 4 Department of Primary Care and Population Health, University of Nicosia Medical School, Nicosia 2408, Cyprus; quattrocchi.a@unic.ac.cy; 5 Microbiology Laboratory, Larnaca General Hospital, Larnaca 6043, Cyprus; mralexandrou@gmail.com (M.A.); pan.papadamou@gmail.com (P.P.); louciapana@gmail.com (L.P.)

**Keywords:** gonorrhoea, *N. gonorrhoeae*, HIV, PLHIV, antibiotics, antimicrobial resistance

## Abstract

**Background:** The rise in antimicrobial-resistant (AMR) strains of *Neisseria gonorrhoeae* is internationally recognised as a critical public health concern, with limited treatment options available. The urgency of this issue prompted the European Centre for Disease Prevention and Control to establish ‘EURO-GASP’ to monitor trends in resistance and address developments. Comprehensive data on AMR strains in people living with HIV (PLHIV) is limited, especially in Cyprus. **Objectives:** To analyse trends in rates of resistant *N. gonorrhoeae* infections and identify any correlations between patient factors that may contribute to such in PLHIV in The Republic of Cyprus. **Methods:** We conducted a retrospective chart review study on *N. gonorrhoea* resistance among PLHIV from the Gregorios HIV reference clinic in Larnaca, Cyprus, between 2015 and 2023. Antimicrobial susceptibility was assessed via disc diffusion or gradient strip method on GC II agar against a non-homogenous panel of antibiotic preparations, based on standard laboratory practice variation. Demographic and clinical data, including antibiograms, treatments and test of cure, were recorded. Statistical analysis was performed using Stata v16, with significance set at *p* < 0.05. The study received approval from the Cyprus National Bioethics Committee. **Results:** A total of 45 isolates from 39 patients were analysed, with 62% of these demonstrating resistance to at least one antibiotic. Resistance rates were not shown to change over time. We identified a statistically significant linear association between a person having a history of an STI and the number of antibiotics which the isolate is resistant to (β = 1.2; *p*: 0.004). Notably, a single isolate demonstrated resistance to ceftriaxone, the first-line treatment currently recommended in both Europe and the United States. This finding is particularly alarming given the critical role of ceftriaxone in the management of gonorrhoea. **Conclusions:** Whilst there has been no increase in resistance rates over time, the detection of ceftriaxone-resistant *N. gonorrhoeae* is a significant public health concern. Given that having a history of an STI makes a person more likely to develop a resistant infection, PLHIV or those who engage in risky sexual behaviours are particularly vulnerable. There is a pressing need to enhance surveillance and implement routine susceptibility testing in Cyprus, given the country’s role as a major international hub for travel and migration. Molecular analysis can further improve our understanding. Additionally, the global public health community must urgently prioritise the development of novel therapeutic agents for the treatment of gonorrhoea.

## 1. Introduction

*Neisseria gonorrhoeae* is one of the most significant causes of sexually transmitted infections (STIs). In 2020, an estimated 82 million new infections occurred globally among individuals aged 15–49 [1]. Across 23 countries in Europe, the incidence rate surged from 4.7 cases per 100,000 persons in population in 2013 to 11.2 cases per 100,000 persons in population in 2022 [2]. Key risk factors for *N. gonorrhoeae* infections include sexual contact with an infected person, previous gonorrhoeal infection, co-infection with other STIs or HIV, having multiple sexual partners and being a man who has sex with men (MSM) [3]. Females are often asymptomatic but may sometimes present with vaginal discharge, dysuria, dyspareunia, bleeding and lower abdominal/rectal pain. In males, symptoms include urethral discharge, itching, dysuria and testicular or rectal pain [3].

*N. gonorrhoeae* has been deemed an urgent threat for antimicrobial resistance (AMR) by the European Centre for Disease Prevention and Control (ECDC) and has even been classified as a ‘superbug’ [4] by the CDC in the United States. First-line treatments began to fail in Europe over a decade ago, largely due to the rise in AMR [5]. Since then, there have been multiple reports of strains with increasing Minimum Inhibitory Concentrations (MICs) towards broad-spectrum cephalosporins [6], which form the cornerstone of treatment today. Although resistance to ceftriaxone itself remains rare, the emergence of such strains underscores the need for optimal therapeutic strategies and eradication. The World Health Organisation (WHO) has expressed concern over the potential evolution of untreatable gonorrhoea, deeming it a public health emergency [7]. In response, the ECDC created the European Gonococcal Antimicrobial Surveillance Programme (Euro-GASP) in 2013 to better ‘inform public health and treatment guidelines’ [8].

Historically, single-dose antibiotics were recommended in the treatment of gonorrhoea; usually penicillin, ciprofloxacin or azithromycin [9]. Currently, third-generation cephalosporins such as ceftriaxone and cefixime are the preferred first-line therapy [10], as resistance to prior agents has exceeded 5% [9]. Ceftriaxone remains the last ‘effective, empiric, first-line’ antibiotic against *N. gonorrhoeae* [11], making monitoring trends in MIC and resistance of paramount importance. Effective antibiotic therapy is crucial as there are no vaccines against the bacterium.

The ECDC recommends that patients with uncomplicated infections of the urethra, cervix or rectum be treated with ceftriaxone 1 g intramuscularly (IM) as a single dose combined with azithromycin 2 g orally, if antimicrobial susceptibility is unknown [10]. Alternatively, patients may be treated with ceftriaxone 1 g IM as a monotherapy, under specific conditions [10]. Test of cure (TOC) is recommended in all cases to ensure the eradication of infection and to identify potential resistance. These treatment recommendations also apply to people living with HIV (PLHIV), who may be at increased risk for resistant infections.

The WHO reports that AMR is caused by factors that include unrestricted access to antimicrobials, inappropriate selection and overuse of antibiotics, and poor-quality antibiotics [12]. Reports state that resistant gonococci have been gradually replacing sensitive bacteria for at least two decades [13]. Proposed mechanisms by which this occurs include enzymatic antimicrobial destruction or modification, target modification or reduced affinity for the antimicrobials and decreased influx/increased efflux of antimicrobials [14]. A single AMR determinant is often not enough to affect clinical outcomes; however, there is a cumulative effect when multiple AMR determinants have been acquired by the microbe, resulting in clinically relevant treatment failure [15]. Given these mechanisms, continuous surveillance is essential to detect emerging resistance and inform treatment strategies.

Understanding the underlying mechanisms of resistance is crucial to appreciating the full clinical threat of AMR. More than 50 chromosomal mutations that affect gonococcal susceptibility to various antibiotics have been identified [16]. For example, one of the most important determinants of resistance for gonorrhoea are mutations in the MtrCDE multidrug efflux pump [17]. This mutation allows for nonspecific resistance against penicillins, macrolides and cephalosporins. Penicillins and cephalosporins target gonococcal penicillin binding protein 2. Mutations in the gene which encodes for this protein, penA, provide increased resistance against these agents [17]. In the context of HIV, these resistance mechanisms are of particular concern, as immunocompromised individuals may require more complex treatment regimes [18]. Moreover, potential drug–drug interactions between antiretrovirals and antibiotics can further complicate treatment, increasing the likelihood of therapeutic failure and the onward transmission of resistant strains.

Rates of AMR vary widely depending on geographic region, access to treatment and the quality of surveillance data. AMR tends to be highest in low-resource and economically disadvantaged countries, where effective therapies are less available. These areas also tend to have a high prevalence of HIV [19]. Studies have shown that PLHIV are at increased risk of AMR across many different bacterial pathogens and drug classes [20]. However, research linking HIV with AMR specific to gonorrhoeal infections is limited. This study aims to identify patterns of AMR *N. gonorrhoeae* infections among PLHIV in the Republic of Cyprus through the descriptive analysis of microbiological and clinical surveillance data. To date, no studies have explored the prevalence or resistance profiles of *N. gonorrhoeae* in this key population within Cyprus, despite the known increased susceptibility and risk of complications in PLHIV. By characterising resistance trends and demographic correlates within this cohort, this study seeks to inform targeted interventions, optimise treatment protocols and contribute to broader antimicrobial stewardship strategies. This study will employ a retrospective analysis of national surveillance datasets spanning the years 2015–2023, focusing on antibiotic susceptibility patterns and co-infection dynamics. As the first study of its kind in the region, the findings are expected to provide valuable insights to support both Cypriot and European public health responses to the rising threat of multidrug-resistant gonorrhoea.

## 2. Methods

We conducted a retrospective chart review study on *N. gonorrhoea* resistance among PLHIV in Cyprus between 2015 and 2023.

Data was extracted from the Gregorios Clinic at Larnaca General Hospital, the national reference clinic for PLHIV in Cyprus. This clinic supports unconditional access to antiretroviral therapy and medical follow-up, regardless of NHS registration or migration status. During the study period, the clinic cared for an average of 913 patients per year (Table 1). Cyprus, an island nation with a population of approximately one million, experiences substantial migratory inflow from four different routes. Shifting population patterns contribute to remarkable diversity in epidemiological trends and pose significant challenges in disease surveillance.

Clinical cases of gonorrhoea were diagnosed based on the culture of swabs taken from the pharynx, urethra and/or rectum depending on the patient’s symptomatology and presenting complaint. Cultures were performed on Thayer-Martin agar (Liofilchem, Roseto degli Abruzzi, Italy), a selective chocolate agar supplemented with haemoglobin, chemical nutrients and antibiotics to inhibit commensal flora [21], whilst still supporting Neisseria growth. Plates were incubated for 24–48 h at 35 °C (+/−2 °C) in an atmosphere containing 5–7% CO_2_ [22]. Colonies with typical morphology were further tested by oxidase test using Kovak’s reagent and by Gram-stain to confirm Gram-negative diplococci. Confirmatory biochemical identification was performed using the API NH kit (bioMérieux, Marcy-l’Étoile, France). Sensitivity was assessed using either the disc-diffusion method or the gradient strip method on GC II agar with haemoglobin and BD BBL IsoVitaleX (Heidelberg, Germany), with the MIC value recorded for the latter. The MIC was defined as the lowest concentration of an antibiotic by which visible growth was inhibited. Not all samples were tested against the same antibiotics—this was dependent upon laboratory protocols at the time and availability of consumables.

Microbiological analysis was performed by the laboratory at the Larnaca General Hospital. Patient records were reviewed using anonymous identification numbers and the following demographic and clinical data was extracted: sex, date of birth, age at diagnosis, sexual orientation, ethnicity, highest-completed educational level (primary, secondary or tertiary studies), date of diagnosis, site of specimen collection, antibiogram results, history of other STIs, co-infection with chlamydia, treatment administered (including dosage) and TOC outcomes, where available. As per standard practice in Cyprus, susceptibility breakpoints were determined based on EUCAST guidelines [23]. Molecular typing was not performed for any isolates as this is not routinely conducted in Cyprus.

This study includes patients diagnosed with *N. gonorrhoeae* between 2015 and 2023 who were confirmed to be HIV-positive. Only individuals aged ≥18 years at the time of diagnosis and residing in Cyprus were eligible for inclusion. All patients were required to have undergone testing via culture at the Gregorios Clinic. Patients were excluded if their records lacked complete susceptibility data or if diagnosis was made via PCR without corresponding culture results.

Descriptive statistics are summarised as the mean ± standard deviation (SD) for continuous variables and as percentages and 95% confidence interval (CI) for categorical variables. To explore factors associated with AMR, simple and multiple linear regression analysis, adjusting for sociodemographic, sexual behaviours and clinical factors, was performed. The outcome variable was represented by the number of active substances to which isolates of *N. gonorrhoeae* were resistant (continuous). All statistical analyses were performed with Stata version 16, and a *p*-value of <0.05 was considered statistically significant.

This study was approved by the Cyprus National Bioethics Committee (ΕΕΒΚ ΕΠ 2023.01.76).

## 3. Results

Between 2015 and 2023, a total of 45 isolates of *N. gonorrhoeae* were retrieved from 39 individual patients (Table 2). The number of cases per study year ranged from 1 to 15, with a mean of 5 (±4) cases per year. There was a peak in cases observed in 2020, followed by a decline in subsequent years. This may be partly attributed to the introduction of the National Healthcare System (NHS) in Cyprus, where patients may have opted to seek routine care through their primary care providers rather than specialised clinics. All cases in this study were isolated from male patients. The mean age at diagnosis was 38.5 years (±9.7). The majority of patients were of Cypriot nationality (77%, 95%CI: 61–89), had attained a tertiary level of education (74%, 95%CI: 58–87) and identified as MSM (82%, 95%CI: 66–92) (Table 2). A prior history of STIs was reported in 22 patients (56%, 95%CI: 40–72), the majority of which (86%, 95%CI: 65–97) were due to syphilis (Table 2). There was no missing demographic data. Among the 45 isolates, 17 (38%, 95%CI: 24–53) were sensitive to all antibiotics tested, while 28 (62%, 95%CI: 47–76) displayed resistance to at least one antibiotic. Specifically, 36% (95%CI: 22–51) were resistant to one antibiotic and 27% (95%CI: 15–42) were resistant to two or more antibiotic preparations (Table 3). For isolates in which the following antibiotics were tested, resistance was seen in approximately 77% (95%CI: 55–92) (n = 17) to gentamicin; 75% (95%CI: 19–99) (n = 3) to ampicillin; 50% (95%CI: 12–88) (n = 3) to ofloxacin; and 39% (95%CI: 17–64) (n = 7) to tetracycline and ciprofloxacin, respectively (Table 3). The prevalence of isolates resistant to at least one antibiotic by sociodemographic characteristic is reported if Supplementary Figure S1.

There is a potential bias here due to the heterogeneity of antibiotic testing. This can be attributed to standard practice variation due to the long period of time over which the data has been extracted. Overall, resistance rates appear to be stable over the study period.

A statistically significant association was found between having a history of an STI and the risk of acquiring an AMR infection. For patients with such a history, the number of antibiotics which they are resistant to, on average, increases by 0.9 (β = 0.93; *p*-value: 0.016). No other significant associations were found between resistance and patient socio-demographic characteristics or clinical variables (Table 4). Notably, history of an STI remained significantly associated with the risk of acquiring an AMR infection also after adjusting for the other covariates (β = 1.2; *p*-value: 0.004).

Treatment information was available for 40 cases. In accordance with guidelines at the time, most patients were treated with either 1g of ceftriaxone monotherapy or a combination of 500mg of ceftriaxone and 1g of azithromycin; in some instances, dual therapy included doxycycline instead. Among the 17 cases where a TOC was performed (either via culture or PCR), all results were negative.

We identified a ceftriaxone-resistant strain of *N. gonorrhoeae* in 2022. This was isolated from a 29-year-old MSM patient born in Cyprus and the infection was acquired locally. The isolate was also resistant to ampicillin, cefotaxime (MIC > 256), tetracycline and gentamicin. The patient was treated with ciprofloxacin based on antimicrobial susceptibility testing and a TOC performed 7 days following completion of therapy was negative.

## 4. Discussion

In this cross-sectional study, we examined resistance patterns of *N. gonorrhoeae* infections amongst PLHIV in the Republic of Cyprus between 2015 and 2023.

Our results yield four important findings: (1) 62% of isolates were resistant to at least one antibiotic for which they were tested; (2) resistance rates remained relatively stable throughout the study period; (3) a ceftriaxone-resistant strain was identified in 2022; and (4) a significant association was found between having a prior history of STIs and an increased risk of acquiring an AMR gonorrhoea infection. This latter finding suggests that because individuals who engage with multiple sexual partners are at higher risk for contracting STIs, said individuals are also at greater risk of AMR infections.

The detection of ceftriaxone resistance is of particular concern. In the most recent EuroGASP Summary of Results in 2020, one isolate out of 3291 displayed ceftriaxone resistance [11], down from three cases in both 2018 and 2019. This decline may reflect the success of dual therapy regimes, as recommended by current European guidelines. Nevertheless, given that ceftriaxone is considered the last reliably effective treatment for gonorrhoea, all cases of resistance warrant urgent public health attention. The identification of such a case in Cyprus in 2022 signals a critical need for continued and enhanced surveillance. It is important to remember that the WHO recommends reconsideration of treatment regimens when resistance rates exceed 5% [24]. If resistance to ceftriaxone begins to approach critical levels, public health authorities will need to consider implementing combination-antibiotic therapy and promote the routine use of molecular diagnostics to guide targeted treatment. Increasing resistance to first-line therapies risks treatment failure, which in turn facilitates ongoing transmission to close contacts. Resistant strains can spread rapidly and as such, it is imperative that the efficacy of ceftriaxone be closely monitored and preserved. The CDC in the United States recommends that patients should be empirically treated with 500 mg of ceftriaxone [25] whilst the ECDC in Europe recommends 1g of ceftriaxone [10]. This difference in dosage may be attributed to higher rates of ceftriaxone resistance in Europe. We observed a notable resistance to older antibiotics: 39% to ciprofloxacin and 15% to penicillin. This contrasts with the rates reported in 2014, where no penicillin resistance was identified in Cyprus [26]. The ECDC has reported constant rates of resistance to ciprofloxacin since 2019, sitting at 58%, but noted a decrease in resistance to cefixime, an alternative first-line treatment [11]. Resistance to gentamicin in our study (77%) is important to note as this antibiotic has been described to be just as efficacious as ceftriaxone in treating gonorrhoea [27].

Our findings align with broader European trends. For example, studies from Austria and Spain have reported increasing azithromycin resistance, rising from 2% to 12% in Vienna between 2013 and 2020 [28], and from 6% to 16% in Catalonia between 2016 and 2019 [24]. Overall, EuroGASP reported rates of azithromycin resistance to be 11%, remaining constant since 2019 [11]. Additionally, in Spain, multidrug-resistant *N. gonorrhoeae* increased from 0.25% to 0.42% [24].

An analysis of trends reported to the ECDC in 2022 [29] has shown a decline in the proportion of *N. gonorrhoeae* isolates that are highly susceptible to third-generation cephalosporins across Europe—a notable shift from the improving susceptibility trends observed since 2016. While ceftriaxone resistance remains rare, sporadic resistant cases have emerged and are often associated with treatment failure, raising concern about the future effectiveness of current regimens. Notably, Cyprus was not included in this study, leaving a critical gap in regional surveillance data. Our findings address this absence by providing the first documented evidence of ceftriaxone resistance in Cyprus, reinforcing the need for the country to be incorporated into broader European monitoring initiatives. In contrast to many of the countries included in this report, our data suggest that the emergence of resistance may already be underway locally.

Pharyngeal and rectal infections are more likely to harbour resistant strains of *N. gonorrhoeae* due to their unique microbial environments which facilitate the exchange of mutations between complex bacteria [16]. Infections of the pharynx and rectum are especially prevalent in MSM populations, who represented 82% of our study cohort and 97% of the PLHIV population reported by the ECDC [11]. Although our data did not show a site-specific increase in AMR, the sample size may have been insufficient to detect such trends.

Our study has several limitations. The small sample size restricted the scope of statistical analysis and may limit the generalisability of our findings. In addition, the lack of standardisation in antibiotic susceptibility testing consistently across cases also reduced internal consistency and comparability. However, data from cases prior to 2015 and from primary care settings was not available for this study but may have strengthened trend analysis and disease burden estimates. Moreover, because our cohort consisted exclusively of PLHIV, the results may not reflect trends in the broader Cypriot population. Furthermore, the ceftriaxone-resistant case that we identified did not undergo further molecular characterisation which limits our ability to understand gonorrhoeal resistance patterns.

Another key limitation is the absence of molecular analysis of resistance determinants in these samples. Nucleic acid-based assays are being increasingly used for the detection of resistance, with more than 50 chromosomal mutations that affect gonococcal susceptibility to antibiotics having been identified [16]. The identification of such mutations in Cypriot isolates, especially in cases of ceftriaxone resistance, might help guide future therapeutic strategies and provide a target for molecular interventions aimed at restoring antibiotic susceptibility.

## 5. Conclusions

The global rise in *N. gonorrhoeae* resistance has not spared Cyprus, where a substantial proportion of clinical isolates demonstrate resistance to multiple antibiotics. The detection of a ceftriaxone-resistant case in this study is particularly alarming, given the limited treatment options currently available. This highlights the urgent need to strengthen surveillance systems and implement routine susceptibility testing which is especially important in Cyprus, a major international hub for travel and migration.

Surveillance should be expanded not only for gonorrhoea but also for other notifiable infections, as current structured programmes are lacking. Molecular analysis especially can improve our understanding of resistance patterns and help track the spread of resistant strains. High-risk populations such as PLHIV should be prioritised in these efforts, as ongoing monitoring in such groups is critical to detect emerging resistance early and guide clinical decision-making.

To better inform public health strategies, future research in Cyprus should include broader population groups to provide a more comprehensive picture of local epidemiology. Larger sample sizes, consistent susceptibility testing protocols and the integration of molecular characterisation will generate more robust data to support clinical and policy-level changes.

Encouragingly, the Ministry of Health is developing a national surveillance system that will integrate clinical and laboratory reporting. Once implemented, this is expected to significantly improve both the scope and quality of reporting. Addressing the growing threat of resistance requires a coordinated global response, including the prioritisation of novel therapeutic development—supported by governmental and private sector funding—to ensure sufficient resources for this challenge.

## Figures and Tables

**Table 1:** Number of *N. gonorrhoeae*-positive isolates amongst total patients receiving treatment at the Gregorios clinic.

	2015	2016	2017	2018	2019	2020	2021	2022	2023	Total
No. of *N. gonorrhoeae* isolates	1	3	3	3	6	15	5	6	3	45
Total patients at Gregorios clinic	568	657	741	836	968	1076	1162	1040	1168	8216

**Table 2:** Characteristics of patients with *N. gonorrhoeae* infection (culture-positive).

Characteristics	Number	% (95%CI)
Male	39	100.0
Female	0	0
Age in years (mean ± SD)	38.5 ± 9.7	
**Sexual orientation**		
Bisexual	4	10.2 (0.3–24)
Heterosexual	3	7.7 (0.2–21)
MSM	32	82.1 (66–92)
**Nationality**		
Bulgarian	1	2.6 (0–13)
Cameroonian	3	7.7 (0.2–21)
Cypriot	30	76.9 (61–89)
Georgian	1	2.6 (0–13)
German	1	2.6 (0–13)
Greek	1	2.6 (0–13)
Liberian	1	2.6(0–13)
Ukrainian	1	2.6 (0–13)
**Educational level**		
Secondary	9	23.1 (11–39)
Tertiary	29	74.4 (58–87)
Unknown	1	2.6 (0–13)
**History of previous STI**		
No	17	44.0 (28–60)
Yes	22	56.0 (40–72)
Hepatitis B	2	9.1 (1.1–29)
Hepatitis B and Chlamydia	1	4.5 (0.1–23)
Syphilis	16	72.7 (50–89)
Syphilis and Chlamydia	2	9.1 (1.1–29)
Syphilis and HPV	1	4.5 (0.1–23)
**Site of isolation (Swab N = 45)**		
Pharynx	9	20.0 (9.6–35)
Pharynx, Urethra, Rectum	2	4.4 (0.5–15)
Rectum	13	28.9 (16–44)
Urethra	21	46.7 (32–62)

MSM: men who have sex with men; STI: sexually transmitted infection; HPV: Human Papilloma Virus.

**Table 3:** *N. gonorrhoea* antimicrobial resistance pattern by year (N = 45 isolates).

	2015	2016	2017	2018	2019	2020	2021	2022	2023	Total
	**N**	**N**	**N**	**N**	**N**	**N**	**N**	**N**	**N**	**N (%, 95%CI)**
**AMPICILLIN**										
R		1	1					1		3 (75, 19–99)
S			1							1 (25, 1–81)
**AZTREONAM**										
R										0 (0, 0–98)
S						1				1 (100, 3–100)
**CEFEPIME**										
R										0 (0, 0–46)
S	1	2		1	2					6 (100, 54–100)
**CEFOTAXIME**										
R								1		1 (7, 0.2–34)
S		2	2		1	1	1	3	3	13 (93, 66–100)
**CEFTAZIDIME**										
R										0 (0)
S		1	1							2 (100, 16–100)
**CEFTRIAXONE**										
R								1		1 (3, 0.1–14)
S	1	3	2	3	5	15	4	2		35 (97, 85–100)
**CEFUROXIME**										
R			1							1 (6, 0.1–29)
S	1	3	1	3	6	2				16 (94, 71–100)
**CIPROFLOXACIN**										
R	1	1	1		2	1			1	7 (39, 17–64)
S		2	1	2	3	1		2		11 (61, 36–83)
**ERYTHROMYCIN**										
R						3		1		4 (13, 3.5–29)
S				2	6	12	4	4		28 (87, 71–96)
**GENTAMICIN**										
R						7	5	5		17 (77, 55–92)
S						2			3	5 (23, 7.8–45)
**OFLOXACIN**										
R	1				1	1				3 (50, 12–88)
S		1		1		1				3 (50, 12–88)
**PENICILLIN G**										
R		2	1			1	1	1		6 (15, 5.9–31)
S		1	1	3	4	14	4	4		31 (79, 64–91)
I					2					2 (5, 0.6–17)
**TETRACYCLINE**										
R		3	2			1		1		7 (39, 17–64)
S	1			3	6	1				11 (61, 36–83)
	**2015**	**2016**	**2017**	**2018**	**2019**	**2020**	**2021**	**2022**	**2023**	**Total**
	**N (%)**	**N (%)**	**N (%)**	**N (%)**	**N (%)**	**N (%)**	**N (%)**	**N (%)**	**N (%)**	**N (%)**
No resistance				3 (100)	4 (67)	7 (47)		1 (17)	2 (67)	17 (38, 24–53)
Resistance to 1 antibiotic		1 (33)	2 (67)		1 (17)	5 (33)	4 (80)	2 (33)	1 (33)	16 (36, 22–51)
Resistance to >1 antibiotics	1 (100)	2 (67)	1 (33)		1 (17)	3 (20)	1 (20)	3 (50)		12 (27, 15–42)

R = resistant; S = sensitive; I = indeterminate.

**Table 4:** Factors associated with antimicrobial-resistant isolates * (simple and multiple linear regression).

	Simple Liner Regression		Multiple Liner Regression	
Variable	Coefficient	*p*-Value	Coefficient	*p*-Value
Age (continuous)	−0.03	0.141	−0.03	0.210
Cyprus nationality	0.28	0.567	0.58	0.233
Sexual orientation				
Heterosexual	Ref			
Bisexual	0.33	0.740	0.48	0.647
MSM	0.49	0.534	0.56	0.457
Year of culture (continuous)	−0.09	0.388	-	-
Culture site				
Pharynx	−0.75	0.093	-	-
Rectum	−0.27	0.517	-	-
Urethra	0.57	0.136	-	-
History of previous STI	0.93	0.016	1.2	0.004

* Outcome variable: number of active substances which *N. gonorrhoea* isolates were resistant to.

## Data Availability

The data presented in this study may be available on request to the corresponding author. The reason for restriction is due to ethico-legal concerns and would require granting of access by the institutional bodies.

## Supplementary Materials

The following supporting information can be downloaded at: https://www.mdpi.com/article/10.3390/antibiotics14060589/s1. Figure S1. Prevalence of antibiotic resistant isolates by sociodemographic characteristics (n = 45).
