# Supplementary material for: Resistance Patterns of Neisseria gonorrhoeae in PLHIV: A Cross-Sectional Study from the Republic of Cyprus, 2015–2023
Source: Antibiotics (Basel). 2025 Jun 7;14(6):589. doi: 10.3390/antibiotics14060589 (PMC12189093; doi:10.3390/antibiotics14060589)

Figure S1. Prevalence of antibiotic resistant isolates by sociodemographic characteristics (n=45)

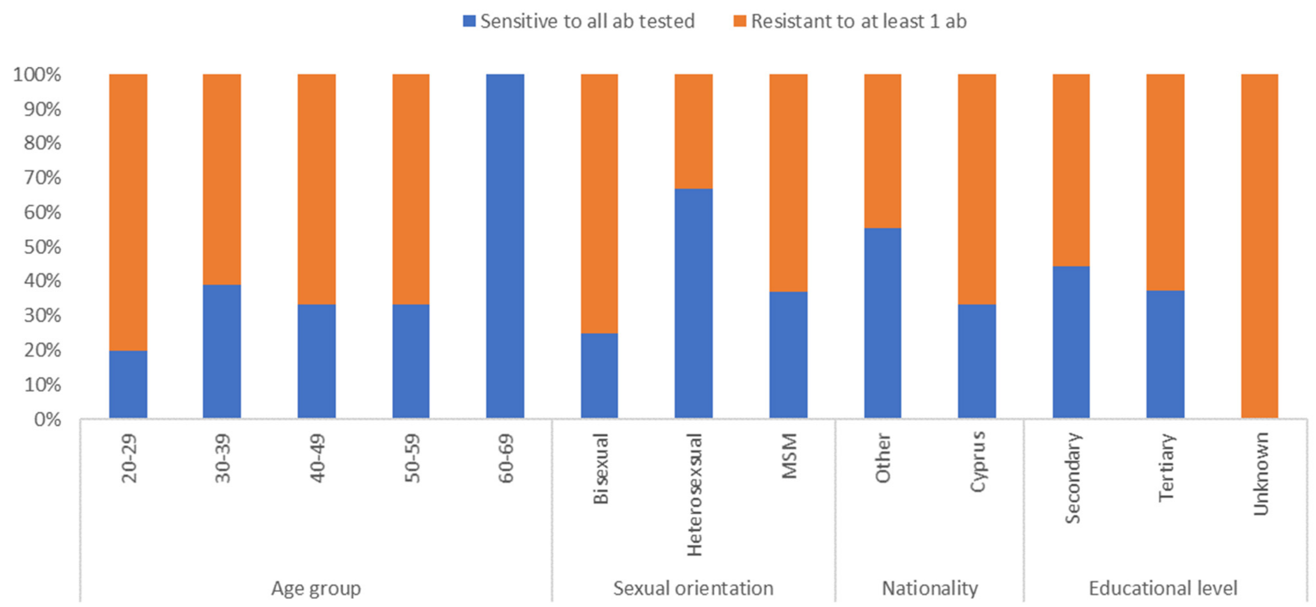

Supplement: Supplementary file 1 [file antibiotics-14-00589-s001.zip › antibiotics-3666348-supplementary.pdf]
